# Salivette, a relevant saliva sampling device for SARS-CoV-2 detection

**DOI:** 10.1080/20002297.2021.1920226

**Published:** 2021-04-30

**Authors:** Monique Melo Costa, Nicolas Benoit, Jerome Dormoi, Remy Amalvict, Nicolas Gomez, Hervé Tissot-Dupont, Matthieu Million, Bruno Pradines, Samuel Granjeaud, Lionel Almeras

**Affiliations:** a Unité Parasitologie Et Entomologie, Département Microbiologie Et Maladies Infectieuses, Institut De Recherche Biomédicale Des Armées, Marseille, France;; b Aix Marseille Univ, IRD, SSA, AP-HM, VITROME, Marseille, France;; c IHU Méditerranée Infection, Marseille, France;; d Centre National De Référence Du Paludisme, Marseille, France;; e CRCM Integrative Bioinformatics Platform, Centre De Recherche En Cancérologie De Marseille, INSERM, U1068, Institut Paoli-Calmettes, CNRS, UMR7258, Aix-Marseille Université UM 105, Marseille, France

**Keywords:** Saliva, COVID-19 diagnosis, coronavirus, SARS-CoV-2

## Abstract

**Background:** The gold standard for COVID-19 diagnosis relies on quantitative reverse-transcriptase polymerase-chain reaction (RT-qPCR) from nasopharyngeal swab (NPS) specimens, but NPSs present several limitations. The simplicity, low invasive and possibility of self-collection of saliva imposed these specimens as a relevant alternative for SARS-CoV-2 detection. However, the discrepancy of saliva test results compared to NPSs made of its use controversial. Here, we assessed Salivettes®, as a standardized saliva collection device, and compared SARS-CoV-2 positivity on paired NPS and saliva specimens.

**Methods:** A total of 303 individuals randomly selected among those investigated for SARS-CoV-2 were enrolled, including 30 (9.9%) patients previously positively tested using NPS (follow-up group), 90 (29.7%) mildly symptomatic and 183 (60.4%) asymptomatic.

**Results:** The RT-qPCR revealed a positive rate of 11.6% (n = 35) and 17.2% (n = 52) for NPSs and saliva samples, respectively. The sensitivity and specificity of saliva samples were 82.9% and 91.4%, respectively, using NPS as reference. The highest proportion of discordant results concerned the follow-up group (33.3%). Although the agreement exceeded 90.0% in the symptomatic and asymptomatic groups, 17 individuals were detected positive only in saliva samples, with consistent medical arguments.

**Conclusion** Saliva collected with Salivette® was more sensitive for detecting symptomatic and pre-symptomatic infections.

## Introduction

In December 2019, a pneumonia outbreak caused by a novel coronavirus, Severe Acute Respiratory Syndrome Coronavirus 2 (SARS-CoV-2), emerged in Wuhan, China. Since then, this disease has spread quickly worldwide causing the Coronavirus Disease 2019 (COVID-19) [1]. In March 2020, this novel coronavirus has been considered as pandemic [2,3]. Breaking the chain of disease transmission was a strategy promoted by the World Health Organization (WHO) to control this health crisis. Extensive screening was recommended to isolate and to treat infected cases. Various modes of transmission were identified, but SARS-CoV-2 spreading via respiratory droplets appeared as the most common [4,5]. Different biological specimens were tested for SARS-CoV-2 detection including upper and lower respiratory tracts and urinary, blood or fecal samples [6,7]. These screening tests are currently carried out by the quantitative reverse-transcription polymerase-chain reaction (RT-qPCR) using mainly nasopharyngeal or oropharyngeal samples [8]. Although nasal/throat swabs remain the gold standard test, this collecting process causes discomfort to patients and may conduct to patient sneezing and coughing, exposing healthcare workers to viral droplets. The airborne dispersal of SARS-CoV-2 represents a considerable risk for healthcare professionals [9]. This uncomfortable sampling method is relatively invasive and nosebleeds might also occur. Moreover, nasal swab is contraindicated for people with blood clotting diseases or deviated septum, for example [10]. Swab sampling is technically not evident to succeed in every case, notably not on very young children or in self-collection [11,12]. Then, this suboptimal collection may reduce test sensitivity, and the recourse to repeat sampling is not infrequent [13].

The validation of a simple and noninvasive sampling method for SARS-CoV-2 diagnosis became demanding. Saliva was then rapidly assessed as an alternative specimen to diagnose COVID-19 [14,15]. Saliva presents numerous advantages, notably low invasive collection, it is easy to handle with the possibility of self-collection, permitting mass testing and preventing exposure of healthcare professionals [16]. Although reports about the screening properties accuracy of saliva for COVID-19 are more and more numerous [17–20], this biological fluid remains dispraised due to its variable diagnostic performance compared to the nasopharyngeal swab (NPS) [21]. The discrepancy observed could be attributed to several factors, such as the heterogeneity of saliva specimens (e.g. spitting saliva, posterior oropharyngeal and deep throat saliva, and drooling collection), the saliva collection methods (e.g. swabs, wide-mouth tube, funnel, soother), or the target population (e.g. hospitalized inpatients, clinically suspected cases, symptomatic, asymptomatic, healthcare workers) [22,23]. To control the ongoing COVID-19 pandemic and to prevent the emergence of new foci of infection, the mass detection of symptomatic and asymptomatic individuals has become essential [24].

In this context, the present study assessed the potential of a new saliva collection system, Salivette®, for COVID-19 diagnosis. This saliva collection system, consisting to moisten a cotton roll, is hygienic, preventing saliva droplets or dripping off the collection tube. This system, dedicated mainly for cortisol measurement, could be used for drug monitoring in saliva [25,26]. The saliva collection with Salivette® was compared to conventional NPS tests using molecular assays in individuals under SARS-CoV-2 investigation. Moreover, the performances of the saliva system were compared with paired NPSs among previously confirmed COVID-19 patients, symptomatic and asymptomatic individuals.

## Materials and methods

*Ethical statement*. The study protocol was reviewed and approved by the Ile de France 1 ethical committee (N°2020-A01249-30 protocol, 06/08/2020). Demographics, clinical data and samples were collected uniquely after the understanding of the study protocol and consent acknowledgement by the participants. A questionnaire on the health status of each participant was completed. All participant information and samples were anonymized prior to use. The sample manipulations were carried out under class II biological safety cabinets MSC-Advantage^TM^ (Thermo Fischer Scientific, Villebon sur Yvette, France).

*Individual recruitment*. During the period of 6 October 2020 to 16 October 2020, individuals admitted to the Institut Hospitalo-Universitaire (IHU) Méditerranée Infection (Marseille, France), for SARS-CoV-2 routine diagnosis, were invited to enroll in the research study. The inclusion criteria were all individuals on demand of SARS-CoV-2 detection using NPS, accepting in parallel, saliva collection. Individuals under 18 years old, non-French speaking, pregnant women and individuals suffering from Gougerot-Sjögren Syndrome were excluded.

*NPS management*. A standard protocol was used for NPSs collection using nasal swabs with viral transport medium (ref. #903,101, Pacific Laboratory Products, Blackburn, Australia). A routine diagnosis protocol was applied for SARS-CoV-2 detection on NPS samples by RT-qPCR [27,28].

*Saliva collection*. Each participant should not have eaten or drunk anything in the 30 minutes prior to saliva collection. Saliva was collected using a cotton roll (Neutral Salivettes®, SARSTEDT, Numbrecht, Germany) under the supervision of a laboratory technician. The cotton roll was directly introduced in the mouth without handling and then kept for 2 min in the mouth’s participant who soaked the cotton by doing circular movements, prior to replace it into the stopper part of the Salivette® tube. The samples were refrigerated on ice at the collection site and stored in these conditions until they arrived in the laboratory. The storing time never exceeded 6 hours.

*Saliva sample preparation*. Salivettes® were centrifuged at 1,500 × g for 2 min at 4°C and retrieved saliva was transferred to 1.5 mL tubes. Two hundred microliters were collected for molecular analysis and the remaining volume was preserved as rescue sample or for others analyses. All samples were stored at −80°C until their use. If the retrieved saliva volume was less than 150 µL, 500 µL of ultra-pure water was loaded at the top of the cotton roll. Salivette® was then, once again, centrifuged at 1,500 × g for 2 min at 4°C and processed as indicated above. The addition of ultra-pure water allowed to retrieve a volume ranging from 200 µL to 500 µL which was sufficient to perform subsequent molecular analysis.

*RNA extraction*. Viral RNA was extracted from 150 µL of the samples (NPS fluids or saliva) using the NucleoMag® Pathogen Isolation kit (MACHEREY-NAGEL GmbH & Co, Düren, Germany). The nucleic acid extraction was fully automated using the KingFisher™ Flex system (ThermoFisher Scientific, Villebon Courtaboeuf, France), within 28 minutes, according to the manufacturer’s instructions. The RNA was recovered in 75 μL of elution buffer and used directly as a template in RT-qPCR for SARS-CoV-2 detection.

*SARS-CoV-2 RT-qPCR*. Five microliters of eluted RNA were tested, targeting the SARS-CoV-2 envelope protein (E)-encoding gene, as previously described [28]. The Enterobacteria phage MS2 (MS2) was added to each sample as internal control [29]. All experiments were performed on a LightCycler 480II instrument (Roche Diagnostics®, Mannheim, Germany). To consider RT-qPCR, the cycle threshold (Ct) value from the MS2 gene should be lower than 32 Ct. For routine diagnosis done on NPSs, samples were classified positive for SARS-CoV-2 when the E primer-probe sets were detected with a Ct value < 35 [30]. When the E primer-probe sets of SARS-CoV-2 were detected with a Ct value > than 35, the NPS samples were classified as negative and non-contagious based on previous studies [30,31]. The same threshold was applied to saliva samples. For the discordant results, uniquely participant classified negatives by NPSs and positives by saliva samples were informed by phone messages, and the participants were invited to perform a retest. Moreover, an examination of the SARS-CoV-2 screening history was done for these participants.

*Human RNase P RT-qPCR*. RT-qPCR using the Human RNase P (HRNP) primers/probe sets were performed as previously described [32], for all saliva samples in order to ensure the quality of the extraction, mainly in the samples with water addition.

*Statistical analyses*. Statistical analyses were performed using the GraphPad Prism software 7.0.0 (GraphPad Software, San Diego, CA). If data verified the required assumptions, parametric tests were applied. If not, nonparametric tests were applied. Frequencies were compared by the Chi-square test and confidence intervals reported. All differences were considered significant at *p < *0.05.

## Results

### Paired comparison of SARS-CoV-2 detection from NPSs and saliva samples

A total of 303 sample pairs of NPSs and saliva samples were collected. The characteristics of the participants are detailed in Table 1. The median age was 40 years (interquartile range, 29–50; range, 18–78 years). One hundred and forty-four individuals (47.5%) were men. Concerning the symptoms, although the median onset (IQR) was 0 day (0–2.5), more than one-third of the participants (n = 104, 34.3%) presented symptoms at the enrolment day. The more common symptoms at presentation were fever (n = 39, 12.9%), myalgia (n = 31, 10.3%) and headache (n = 24, 7.9%), corresponding to influenza symptoms, followed by cough (n = 21, 6.9%) and anosmia/ageusia (n = 20, 6.6%) which were frequently reported in Covid-19 clinical diagnosis criteria [33,34].

**Table 1.** Characteristics of participants under investigation for COVID-19 diagnosis by paired NPSs and saliva samples

	Overall	Follow-up*	Symptomatic	Asymptomatic
Participants, n (%)	303	30 (9.9%)	90 (29.7%)	183 (60.4%)
Age (years), median (IQR)	40 (29–50)	45 (34.5–58.8)	41 (28.3–50.0)	39 (28.5–49.5)
Male, n (%)	144 (47.5%)	16 (53.3%)	35 (38.9%)	93 (50.8%)
Onset of symptoms before the test (days), median (IQR)	0 (0–2.5)	8 (6–11)	3 (1–5)	/
Symptoms at presentation, n (%)	104 (34.3%)	14 (46.7%)	90 (100%)	0 (0.0%)
Cough, n (%)	21 (6.9%)	3 (10.0%)	18 (20.0%)	/
Sore throat, n (%)	16 (5.3%)	1 (3.3%)	15 (16.7%)	/
Runny nose, n (%)	18 (5.9%)	0 (0.0%)	18 (20.0%)	/
Anosmia/Ageusia, n (%)	20 (6.6%)	6 (20.0%)	14 (15.6%)	/
Diarrhea, n (%)	6 (2.0%)	0 (0.0%)	6 (6.7%)	/
Myalgia, n (%)	31 (10.3%)	3 (10.0%)	28 (31.1%)	/
Fever, n (%)	39 (12.9%)	3 (10.0%)	36 (40.0%)	/
Tiredness, n (%)	12 (4.0%)	3 (10.0%)	9 (10.0%)	/
Headache, n (%)	24 (7.9%)	0 (0.0%)	24 (26.7%)	/
Others, n (%)	3 (1.0%)	1 (3.3%)	2 (2.2%)	/

* Previously tested positively for SARS-CoV-2 by RT-qPCR on NPSs. Abbreviations: IQR, interquartile range; NPS, nasopharyngeal swab; SARS-CoV-2, severe acute respiratory syndrome coronavirus 2; SD, standard deviation.

**Figure 1.**
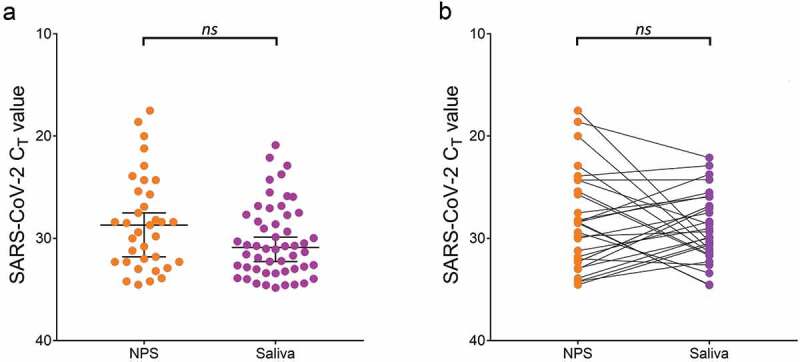
Comparison of Ct values from NPSs and saliva samples. (**A**) All positive NPSs (n = 35) and saliva samples (n = 52) were compared using a Mann–Whitney test (*p = *0.097). Bars represent the median and 95% CI. (**B**) Paired positive samples (n = 29), represented by the connecting lines, were compared by a Wilcoxon test (*p = *0.761)

Paired sample analyses revealed that the positive rate of SARS-CoV-2 screening by RT-qPCR for NPSs and saliva samples were 11.6% (n = 35) and 17.2% (n = 52), respectively. Among them, 29 participants were classified as positives using both specimens, and 6 and 23 were classified positively for SARS-CoV-2 only by NPSs or saliva, respectively (Table 2). The comparison of Ct values between NPSs and saliva samples were not significantly different either if all positives samples (*p > *0.05, Mann–Whitney test, Figure 1A) were considered, or if paired positive samples (*p > *0.05, Wilcoxon test, Figure 1B) were taken into account.

**Table 2.** Comparison of the RT-qPCR detection of SARS-CoV-2 between NPSs and saliva samples

Saliva samples	NPSs		Total
	Positive	Negative	
Positive	29	23	52
Negative	6	245	251
Total	35	268	303
Agreement (%)	90.4%		
Cohen’s κ ^#^	0.652 (Substantial)		
Sensitivity (%)	82.9%		
Specificity (%)	91.4%		

^#^Coefficient of agreement, the agreement level is indicated into brackets, as previously defined [35]. NPS, nasopharyngeal swab.

To determine the screening test performance of RT-qPCR on saliva samples, the results from the NPSs were used as reference. The sensitivity and specificity of saliva samples were, respectively, of 82.9% and 91.4% (Table 2). The assessment of concordance of RT-qPCR results between paired specimens revealed an agreement of 90.4% with a Cohen’s κ coefficient of 0.652 corresponding to a substantial agreement of the data [35].

### Performances of screening tests according to clinical history and symptoms

Among the enrolled volunteers, 30 participants were previously positively tested for SARS-CoV-2 by RT-qPCR on NPSs and came to follow their viral load (follow-up group, n = 30; 9.9%). The delay between the previous NPS SARS-CoV-2 positive tests and the present sampling ranged from 4 to 10 days. The remaining participants were separated into mildly symptomatic (n = 90; 29.7%) and asymptomatic (n = 183; 60.4%) groups (Table 1). No significant differences were noted between the ages (*p = *0.189, Kruskal–Wallis test) or the gender (*p = *0.489, df = 2, Chi-square test) among the three groups. Conversely, the median days post-symptoms onset was significantly longer for the follow-up group compared to the symptomatic group (*p < *0.001, Mann–Whitney test). The main registered symptoms at presentation were anosmia/ageusia (n = 6; 20.0%) for the follow-up group, and fever (n = 36; 40.0%) and myalgia (n = 28; 31.1%) for the symptomatic group. The positive rate of SARS-CoV-2 screening by RT-qPCR for NPSs and saliva samples was, respectively, 20.0% (n = 6) and 26.7% (n = 8) for the follow-up group (*p > *0.05, Chi-square test), 24.4% (n = 22) and 30.0% (n = 27) for the symptomatic group (*p > *0.05, Chi-square test), and 3.8% (n = 7) and 9.3% (n = 17) for the asymptomatic group (*p < *0.05, Chi-square test, Table 3). In all groups, the positive rate was higher in saliva samples than in NPSs, and this difference was significantly increased for the asymptomatic group.

**Table 3.** Comparison of the RT-qPCR detection of SARS-CoV-2 between NPSs and saliva samples according to participant status

Saliva samples	NPSs						Total
	Follow-up*		Symptomatic		Asymptomatic		
	Positive	Negative	Positive	Negative	Positive	Negative	
Positive	2	6	20	7	7	10	52
Negative	4	18	2	61	0	166	251
Total	6	24	22	68	7	176	303
Agreement (%)	66.7%		90.0%		94.5%		
Cohen’s κ ^#^	0.074 (Slight)		0.749 (Substantial)		0.559 (Moderate)		
Sensitivity (%)	33.3%		90.9%		100%		
Specificity (%)	75.0%		89.7%		94.3%		

*Previously tested positively for SARS-CoV-2 by RT-qPCR on NPSs. ^#^Coefficient of agreement, the agreement level is indicated into brackets, as previously defined [35]. NPS, nasopharyngeal swab.

In the follow-up group (n = 30), discordant results were obtained for one-third (n = 10) of the tested specimens leading to low sensitivity (50.0%) and specificity (75.0%) for saliva samples compared to NPSs (Table 3). Consequently, the proportion of agreement was weak (66.7%; Cohen’s κ coefficient of 0.074, slight agreement). Interestingly, among the four participants classified as positives uniquely by NPSs tests, three were detected from saliva samples with Ct values ranging from 35.1 to 36.9 (Additional File 1). Conversely, among the six participants classified as positives uniquely by saliva tests, one obtained a Ct of 36.58 using NPSs tests. Finally, only one participant was absolutely not detected by saliva specimens, whereas five were failed to be perceived by NPSs (Ct > 38).

For the symptomatic group, the sensitivity and specificity reached, respectively, 90.9% and 89.7% for saliva samples compared to NPSs (Table 3). The accuracy between the two specimens revealed an agreement of 90.0% with a Cohen’s κ coefficient of 0.749 corresponding to substantial agreement. Discordant results were obtained for nine participants. Among them, SARS-CoV-2 was detected uniquely in NPSs of two participants and in saliva samples from seven individuals (Additional File 1). Among the seven symptomatic participants positively tested by saliva samples, follow-up information was collected for three of them. Two performed a new NPS (7 to 9 days later) that confirmed their positivity (Ct values of 31.3 and 32.0). The last one indicated that his/her partner was positively detected by NPSs 3 days before his/her sampling. No information was collected for the remaining participants of this group.

For the asymptomatic group, a sensitivity and specificity of 100% and 94.3%, respectively, was obtained for saliva samples compared to NPSs (Table 3). The agreement proportion reached to 94.5% with a Cohen’s κ coefficient of 0.559 corresponding to moderate agreement. Ten asymptomatic participants were detected SARS-CoV-2 positive uniquely in saliva samples (Ct values ranging from 20.9 to 34.0). For three of those ten participants, influenza symptoms appeared three or four days after the positive saliva test (Additional File 1). Two out of those three performed a new NPS test and were both positives (Ct values of 21.6 vs 32.8 three days before in saliva and 23.9 vs 20.9). Among the remaining seven of those ten participants, the partners of two participants were tested positive by NPSs two and six days before. No information was obtained from the five remaining participants positively detected by saliva samples. The analyses of clinical data and phone contacts supported that more than half of the discordant results (n = 18; 62.1%) were likely true positives. The absence of complementary information from the remaining 11 participants did not allowed us to adjudicate on their infectious status.

If we consider as true positive any participants with either a NPS or a saliva positive tests, the total number of positives samples was 58, corresponding to a positive rate of 19.1% (Table 4). In these conditions, the overall proportion of agreement of SARS-CoV-2 screening for saliva (98.0%, Cohen’s κ coefficient of 0.933, almost perfect agreement), was significantly more accurate than for NPS (92.4%, Cohen’s κ coefficient of 0.711, substantial agreement; *p < *0.003, Chi-square test). The positive percent agreement (PPA, similar to sensitivity) was found significantly higher for saliva (89.7%) compared to NPS (60.3%, *p < *0.003, Chi-square test). By detecting 17 more cases than NPS, saliva obtained a clear higher performance for SARS-CoV-2 detection.

**Table 4.** Comparison of the RT-qPCR detection of SARS-CoV-2 between NPSs and saliva samples to a reference that considers a person to be positive if one of his or her samples is positive

	NPSs		Saliva		Total	Chi-square test	95% CI
	Positive	Negative	Positive	Negative			
Positive	35	0	52	0			
Negative	23	245	6	245			
Total	58	245	58	268	303		
Agreement (%)	92.4%		98.0%			*p < *0.003	[−0.093; −0.019]
Cohen’s κ ^#^	0.711 (Substantial)		0.933 (Almost perfect)				
PPA (%)	60.3%		89.7%			*p < *0.001	[−0.458; −0.128]

^#^Coefficient of agreement, the agreement level is indicated into brackets, as previously defined [35]. CI, confidence interval; NPS, nasopharyngeal swab; PPA, positive percent agreement.

### RNA detection in diluted saliva samples

To control whether the addition of ultra-pure water to saliva samples could be detrimental for RNA detection, a comparison of Ct values from HRNP between the 34 saliva samples with addition of ultra-pure water, and 269 remaining saliva samples was performed. No HRNP amplification was obtained for one saliva sample with water addition, whereas, the failing of PCR products in four saliva samples, which did not required water addition, was observed. HRNP Ct values from saliva samples with water addition were significantly lower than those without water addition (*p < *0.003, Mann–Whitney test, Additional File 2A). Nevertheless, among the 34 saliva samples filled up with ultra-pure water, SARS-CoV-2 was detected in five samples (Ct values ranging from 29.9 to 34.0). HRNP products were obtained for these five samples (Ct values ranging from 26.7 to 33.2). Among them, one participant, positively detected for SARS-CoV-2 using saliva (Ct value: 29.9), was also corroborated by the NPS specimen (Ct value: 33.9). These results indicated that, despite water addition decreased detection of human RNA cellular control, SARS-CoV-2 detection was not compromised by the saliva dilution. The non-access of NPS specimens did not allowed us to assess HRNP detection, and then to conclude whether miss-detection in NPSs was attributed to improper sampling (no PCR product) or to insufficient local viral titers (lower than detection limit).

Interestingly, the HRNP was detected in the 52 SARS-CoV-2 saliva samples and no significant difference of HRNP Ct values was observed between positives and negatives SARS-CoV-2 saliva samples (*p > *0.05, Mann–Whitney test, Additional File 2B). Moreover, HRNP was detected in the six saliva samples (Ct values ranging from 29.9 to 33.9), for which respective NPS specimens were found SARS-CoV-2 positives. The mis-detection of the coronavirus in these saliva samples was not due to the RNA extraction impairment.

## Discussion

Although salivary tests for SARS-CoV-2 detection are currently used in routine diagnostic laboratories for patient investigations in several countries from Asia, America and Europe [12,36–38], this tool remains under progress for validation in France [39]. The insufficient sensitivity of salivary tests based on molecular approaches (e.g. RT-qPCR, LAMP-PCR) compared to NPSs, remains the main bottleneck to consider these tests for mass application by authorities [40–42]. Nevertheless, an increase in the sensitivity of saliva specimens compared to NPSs is described in accumulative works conducted in hospitalized patients [43,44], and in symptomatic [45] or asymptomatic individuals [17,44], which led to questions about the saliva performance for detection of SARS-CoV-2. The multitude of saliva sampling methods, plus the inter-individual variability due to non-compliant saliva collection, prevented the establishment of a clear conclusion [46]. The establishment of a procedure with a simple system for saliva collection became compulsory.

In the present study, Salivette® devices were chosen to standardize saliva collection. For an accurate comparison of saliva and NPS specimens, the same extraction method and RT-qPCR systems, with identical experimental control and validation criteria, were used. Overall, the present study confirmed the relevance of the saliva sampling for SARS-CoV-2 detection in the 303 individuals under investigations, with a concordance to NPSs exceeding 90%, that was among the more efficient methods [22]. Nevertheless, among the 58 participants classified as SARS-CoV-2 positive either by NPS and/or saliva tests, discordant paired detection represented half of them (n = 29). Most of the discordant pairs were detected positive only in saliva samples (79.3%, 23/29). Investigations based on clinical history analyses and patient phone contacts revealed a confirmation or supportive data of SARS-CoV-2 infection for 60.9% of them (14/23). The lack of data prevented conclusion on the SARS-CoV-2 *status* of the remaining participants. If any participant with either an NPS or a saliva positive tests is considered a true positive, as it is frequently done [47,48], the PPA was incontestably significantly higher for saliva than in NPS. This was consistent with previous works reporting an increased sensitivity of saliva specimens [44,49–51]. The absence of significant differences in SARS-CoV-2 Ct values, when all positives or paired positive specimens were compared, confirmed that the increased sensitivity could not be attributed to an improved detection of the virus in saliva. The inconsistency of NPS sampling that induces false negatives seems to be a recurrent phenomenon especially in patients with a low viral load [52], frequently reported during serial testing [13,53,54] or in early stage of infection [55].

To limit false negative detection, RNA template or synthetic RNA were proposed as external controls for the proper extraction and amplification [56]. However, these external RNAs did not insure a proper sample collection nor the integrity of the RNA sample [28,32,57], and the use of a human RNA cellular control was proposed. The US CDC proposed to use HRNP as a control of proper sampling, sample preservation and extraction [58]. In our study, we did not have access to the NPS samples of the routine diagnosis, then it was not possible to control whether the discordance between NPS and saliva specimens was attributed to an RNA integrity failure or a mis-detection of the virus. Conversely, the amplification of the HRNP in the saliva samples from participants classified positive by NPS tests, underlined that the mis-detection seems more attributed to an absence or a sub-detection of the virus rather than a problem of saliva collection. The systematic used of human RNA cellular control is mandatory if the sampling sources are to improve results interpretation.

The risk to fail in saliva sampling seems less frequent than for NPSs. In a recent study assessing the efficiency of Salivette® for screening SARS-CoV-2 hospitalized cases, 12.2% (6/49) of patients were excluded for failing saliva volume [48]. Sialadenitis, an acute inflammation of salivary glands reported in COVID-19 patients, could lead to a decrease of salivary flow compromising saliva collection [59]. We observed an insufficient volume of saliva in 11.2% of the samples (n = 34). The large majority of these individuals (29/34; 85.3%) were tested SARS-CoV-2 negative in saliva, suggesting that, here, lower salivation could not be attributed to viral infection. Concerning the human cellular RNA control, adding water did not impair the detection of the HRNP. Although significant differences of HRNP Ct values were noticed between diluted and not diluted saliva samples, the SARS-CoV-2 detection in five saliva specimens underlined that viral detection does not seem altered by diluting saliva. The water addition at the top of the cotton roll allowed to recover most of the saliva samples (n = 33/34, 97.1%), for which HRNP was detected. Finally, water addition did not compromised RNA detection and the RNA integrity control allows to reduce false negative detection. To reduce the proportion of samples for which the saliva volume retrieved were insufficient, a better explanation of its use with a short video describing the proper use of Salivette would be helpful.

The saliva tests were also compared to NPSs according to the clinical history and symptomatology of the individuals. A weak agreement was observed in the follow-up group. As patients from this group were convalescent, their viral charge was decreasing [14], and then mis-paired viral detection could have occurred. Among them 10 discordant detections, 4 could be considered as non-contagious based on Ct values from paired samples (35< Ct < 38) [30]. The decrease in the viral load after the first week after the onset of symptoms was consistent with previous findings in NPSs [60,61], as well as in saliva samples [62,63].

To assess the COVID-19 spreading, an accurate identification of infectious patients regarding SARS-CoV-2 among symptomatic and asymptomatic individuals is essential. Here, although the proportion of agreement between NPS and saliva specimens exceeded 90%, numerous individuals (n = 17) were detected only in saliva samples. Saliva collected with Salivette® appeared more sensitive for detecting symptomatic and pre-symptomatic infections. Complementary experiments in others routine diagnosis sites are required to validate these results. Previously, the used of Salivette® devices on COVID-19 hospitalized patients reported a sensibility and specificity of 100% and 97.2%, respectively, when NPSs were used as references [48]. Salivette® presents several advantages, such as a better acceptation than NPSs for sample collection, notably in children. Moreover, the inoffensiveness of sampling allows to perform daily collection from hospitalized patients to follow the viral titer. It will be interesting to assess whether saliva collection with Salivettes could improve the detection of antibody responses against SARS-CoV-2 viral antigens, as described previously [48], in order to look for past infection with SARS-CoV-2 or to monitor serological response after vaccination. The detection of the influenza virus [64] or respiratory syncytial virus [65], in patient saliva has already been reported. Replacing throat swabs, nasopharyngeal or bronchoalveolar aspirates by saliva collection with the Salivette system could represent an important benefit, ensuring non-invasive sampling and self-collection.

## Conclusion

When Europa is heading into the winter months and the risk of influenza symptoms is expected to increase that it could be difficult to distinct COVID-19 from non-COVID-19 individuals based on clinical symptoms. The setup of a screening system allowing rapid self-collection and accurate SARS-CoV-2 detection should be helpful in isolating infectious individuals. The present work confirmed that the saliva test is a reliable alternative to NPSs for SARS-CoV-2 detection. Saliva specimens showed significantly higher performance for detection of the virus in symptomatic and asymptomatic individuals. The use of a standardized saliva collection device and routine sample integrity testing with a human cellular RNA should reduce inter-sample variation and this combination should validate the use of saliva as a diagnostic tool for SARS-CoV-2. A short-time validation of these guidelines by another SARS-CoV-2 screening center is compulsory.

## Supplementary Material

Supplemental Material

## Figures and Tables

**Figure:**
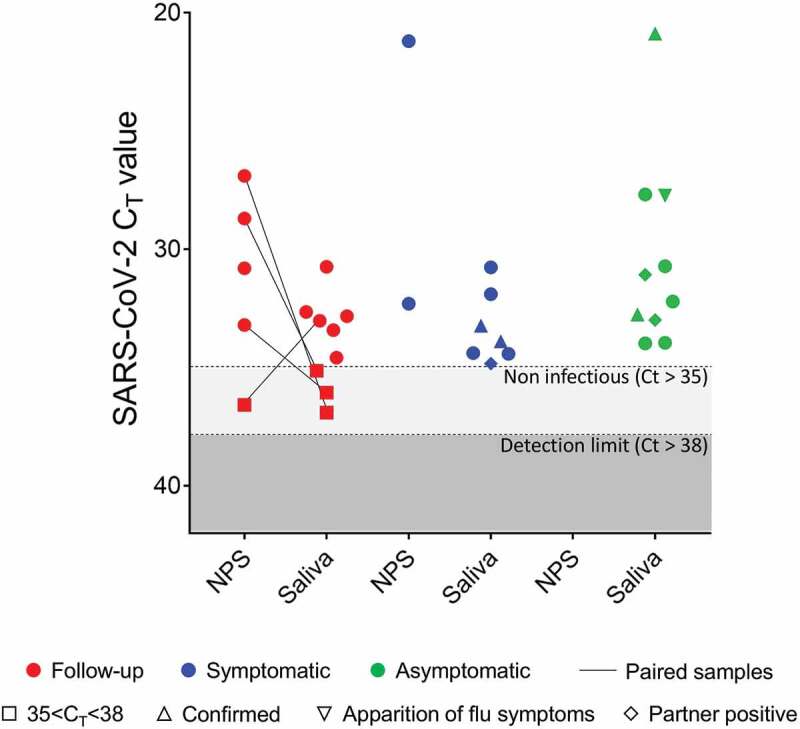


**Figure:**
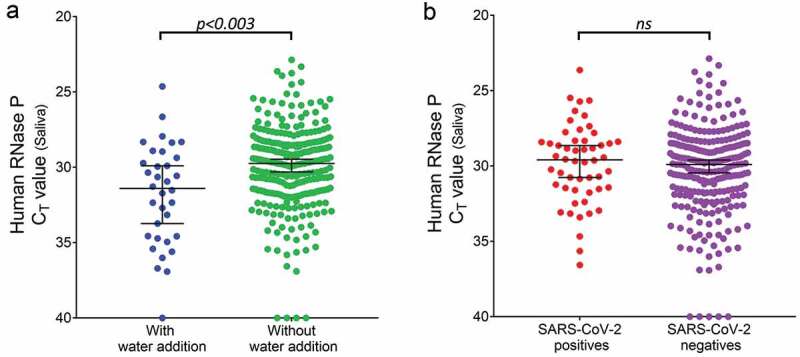


## List of abbreviations

NPS: nasopharyngeal swab; PCR: Polymerase Chain Reaction; Ct: Cycle threshold; RT-qPCR: Reverse transcription quantitative real-time PCR; SARS-CoV-2: Severe Acute Respiratory Syndrome Coronavirus 2; COVID-19: Coronavirus Disease 2019: PPA: positive percent agreement.

## Notes

**Additional File 1**. SARS-CoV-2 Ct values from discordant results of paired NPS and saliva samples according to clinical history or symptoms. Paired samples with Ct > 38 were not presented. Paired samples detected at a non-infectious level (35 < Ct < 38) are indicated by squares. Triangles represent participants with influenza symptoms apparition with (arrow up) or without (arrow down) confirmed SARS-CoV-2 positive test. The participants for who his/her partner were declared SARS-CoV-2 positive few days before are represented by a diamond.

**Additional File 2**. Consequences of water addition to saliva samples on RNA detection. (A) Comparison of human RNase P Ct values between saliva samples with (n=34) and without (n=269) water addition (p < 0.003, Mann–Whitney test). (B) Comparison of human RNase P Ct values between positive (n=52) and negative (n=251) SARS-Cov-2 saliva samples (p>0.05, Mann–Whitney test). Bars represent the median and 95% CI.
