# Supplementary material for: Salivette, a relevant saliva sampling device for SARS-CoV-2 detection
Source: J Oral Microbiol. 2021 Apr 30;13(1):1920226. doi: 10.1080/20002297.2021.1920226 (PMC8098750; doi:10.1080/20002297.2021.1920226)
Supplement: Supplemental Material [file ZJOM_A_1920226_SM6222.zip › Supplementary files/Supplementary Legend.docx]

**Supporting information files**

**Additional File 1.** SARS-CoV-2 Ct values from discordant results of paired NPS and saliva samples according to clinical history or symptoms. Paired samples with Ct > 38 were not presented. Paired samples detected at a non-infectious level (35 < Ct < 38) are indicated by squares. Triangles represent participants with influenza symptoms apparition with (arrow up) or without (arrow down) confirmed SARS-CoV-2 positive test. The participants for who his/her partner were declared SARS-CoV-2 positive few days before are represented by a diamond.

**Additional File 2.** Consequences of water addition to saliva samples on RNA detection. (**A**) Comparison of human RNase P Ct values between saliva samples with (n=34) and without (n=269) water addition (*p<*0.003*,* Mann-Whitney test). (**B**) Comparison of human RNase P Ct values between positive (n=52) and negative (n=251) SARS-Cov-2 saliva samples (*p>*0.05*,* Mann-Whitney test). Bars represent the median and 95% CI.
