# Supplementary figures and images for: Salivette, a relevant saliva sampling device for SARS-CoV-2 detection
Source: J Oral Microbiol. 2021 Apr 30;13(1):1920226. doi: 10.1080/20002297.2021.1920226 (PMC8098750; doi:10.1080/20002297.2021.1920226)

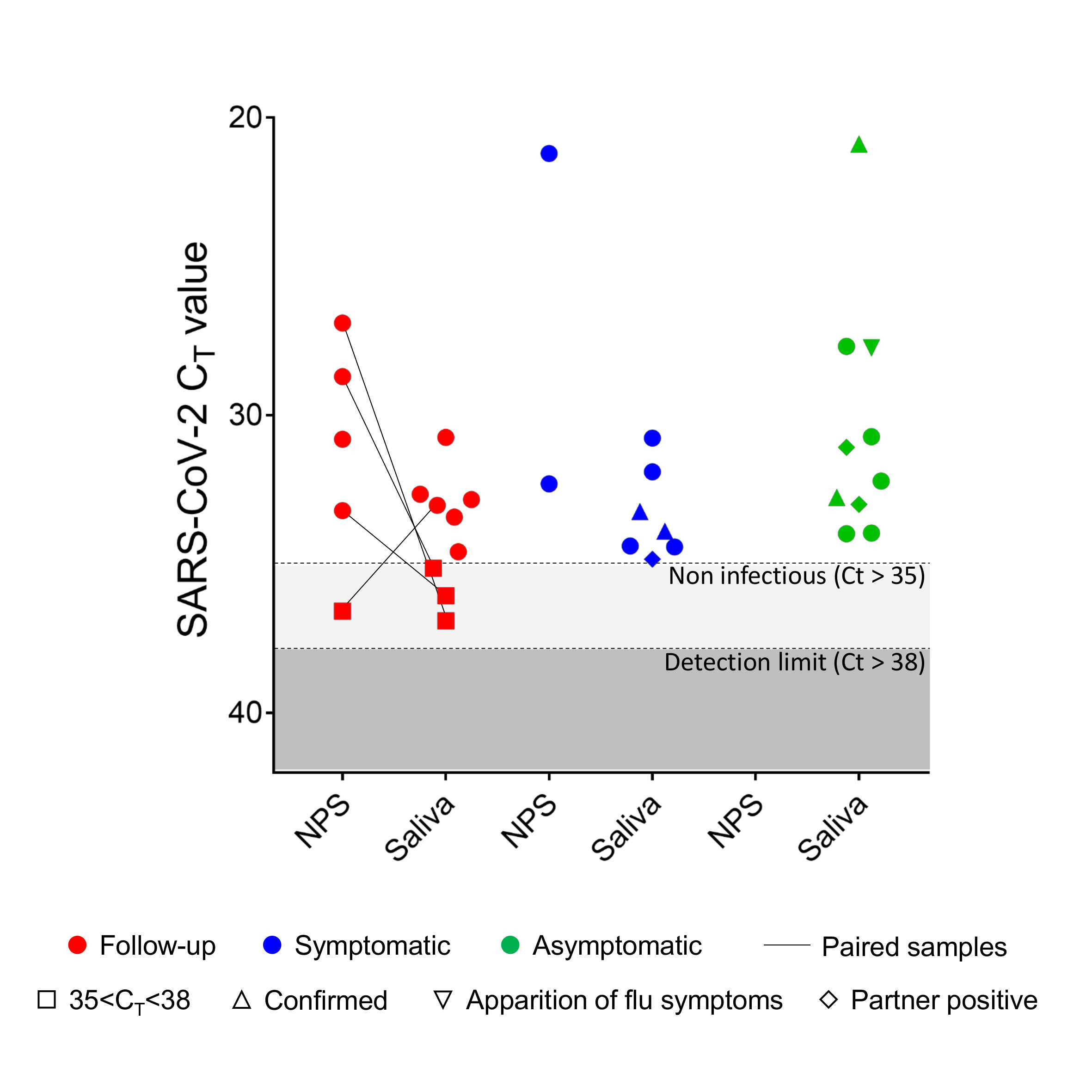

Supplement: Supplemental Material [file ZJOM_A_1920226_SM6222.zip › Supplementary files/Additional_File_1.jpg]

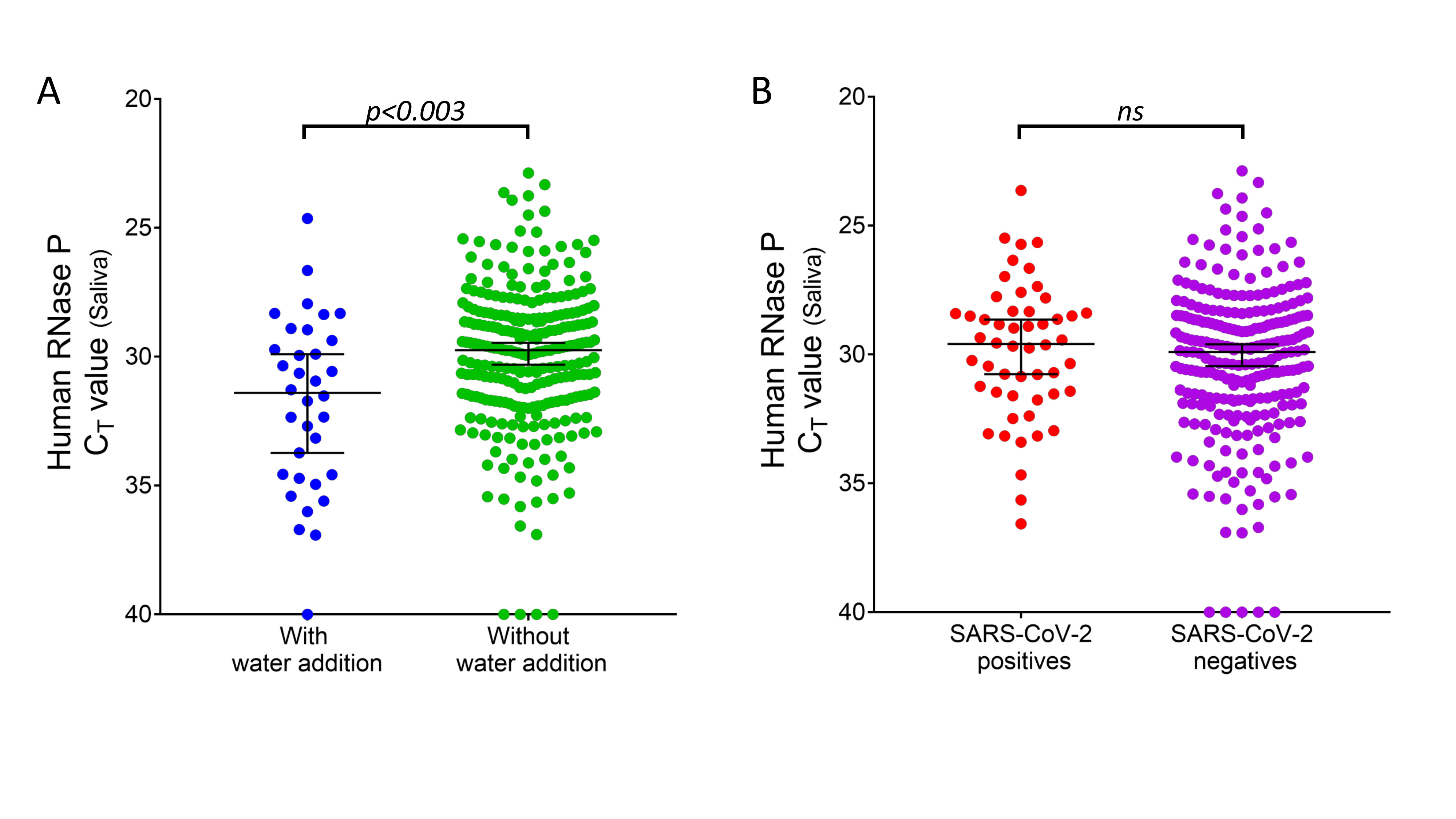

Supplement: Supplemental Material [file ZJOM_A_1920226_SM6222.zip › Supplementary files/Additional_File_2.jpg]
